# ATG13 is involved in immune response of pathogen invasion in blood clam *Tegillarca granosa*

**DOI:** 10.3389/fvets.2023.1141284

**Published:** 2023-03-02

**Authors:** Yuling Wu, Guosheng Liu, Zengpeng Li, Mingliang Chen, Qin Wang

**Affiliations:** ^1^State Key Laboratory Breeding Base of Marine Genetic Resources, Third Institute of Oceanography, Ministry of Natural Resources, Xiamen, China; ^2^School of Life Science, Xiamen University, Xiamen, China; ^3^Co-innovation Center of Jiangsu Marine Bio-Industry Technology, Jiangsu Ocean University, Lianyungang, China; ^4^School of Marine Biology, Xiamen Ocean Vocational College, Xiamen, China

**Keywords:** *Tegillarca granosa*, ATG13, autophagy, vibrio infection, LPS

## Abstract

Mammalian autophagy-related gene 13 (ATG13) is a vital component of the ATG1 autophagy initiation complex which plays an essential role in autophagy. However, the molecular function of ATG13 in pathogen defense in invertebrates is still poorly understood. In this study, the full-length cDNA sequence of blood clam *Tegillarca granosa* ATG13 (TgATG13) was obtained, which was 1,918 bp in length, including 283 bp 5' UTR, 252 bp 3' UTR and 1,383 bp open reading frame (ORF) encoding 460 amino acids. Phylogenetic analysis revealed that TgATG13 had the closest relationship with that of *Crassostrea Virginica*. Quantitative real-time PCR results showed that the transcript of TgATG13 was universally expressed in various tissues of blood clam, with the highest expression level in hemocytes. The expression level of TgATG13 was robustly increased after exposure of both *Vibrio alginolyticus* and LPS. Fluorescence confocal microscopy further showed that TgATG13 promoted the production of autophagosome. In summary, our study demonstrated that TgATG13 was involved in the immune regulation of blood clam during pathogen invasion, deepening our understanding of the innate immune mechanism of blood clam.

## 1. Introduction

Blood clam (*Tegillarca granosa*) has become an important economic product of global aquaculture. Inhabited in mudflats, blood clam is a type of classic shellfish which is characterized by inactivity and strong resistance. It was reported that as the seafood treasure, blood clam can enhance human immunity and inhibit tumor activity (1–3). Over the past two decades, blood clam had also been an attractive model in innate immunology and marine toxicology (4, 5).

Vibriosis, one of the most common diseases in marine shellfish, causes massive mortality and huge economic loss of shellfish industry, which severely hinders the development of marine shellfish aquaculture (6–9). Recently, diseases of blood clam have become increasingly prominent worldwide, resulting in the frequently massive death. Dahanayake et al. (10) isolated 32 *Vibrio* strains from blood clam, including 4 strains of *Vibrio parahaemolyticus* and 11 strains of *Vibro alginolyticus*, suggesting that *Vibrio* was a main pathogen that infects blood clam.

Autophagy is an intracellular self-degradative pathway which removes unnecessary cytoplasmic constituents by lysosome-dependent degradation. Previous studies have demonstrated that autophagy is highly conserved in eukaryotes. Autophagy plays important roles in a wide variety of physiological and pathophysiological processes, such as cardiovascular and liver diseases, metabolic disorders aging and cancer (11–13). Moreover, it was also reported that autophagy is involved in the pathogen defense (14, 15). Autophagy process is mediated by the double-membrane autophagosome. There are more than 50 genes related to the formation of autophagosomes, more than 30 of which are autophagy-related genes (ATG) (16). ATG proteins can impose great effect on the initiation, extension, maturation and degradation of autophagy (17). As an important autophagy factor, ATG13 is a key component of the multimeric ULK complex which is required for the autophagosome biogenesis. Besides, ATG13 is also one of the key targets of mTOR kinase signaling pathway. This pathway regulates autophagy through two ways: one is phosphorylation of ATG13 and ULK1, the other one is to regulate the formation of ATG13-ULK1-RB1CC1 complex (18–20). Asynchronously, ULK1-ATG13, as the most upstream autophagy initiation protein, is phosphorylated by rapamycin complex 1 (mTORC1) and AMP-activated protein kinase (AMPK) to induce autophagy. However, their phosphorylation regulation and function in mitosis and cell cycle remain unclear (18). Li et al. (21) found that CDK1 kinase-induced phosphorylation of ULK1-ATG13 promotes mitosis autophagy and cell cycle progression. In addition, double knockout of *ULK1* and *ATG13* genes could block the cell cycle process and significantly reduce the proliferation effect of cancer cells. These results establish a bridge between autophagy and mitotic core mechanisms, and elucidate the positive role of ULK1-ATG13 protein complex and its phosphorylation in the regulation of mitotic autophagy (21).

Although autophagy pathway is well-characterized in vertebrates, its functional study in invertebrates, especially in shellfish, is very limited. Picot et al. (22) identified for the first time the autophagy pathway network which includes 35 ATG proteins in Pacific oyster (*Crassostrea gigas*). *C. gigas* ATG10 participates in the immune response against poly (I:C) by regulation of autophagosome biogenesis (23). Dang et al. (24) found that autophagic signaling was activated when *Crassostrea hongkongensis* was infected by *V. parahaemolyticus*, indicating that autophagy in blood lymphocytes is an important way of host defense against vibrio infection in mollusks. So far, the autophagy pathway in *T. granosa* is poorly understood. In this study, we identified an ATG13 homolog gene in *T. granosa*, and explored its role in pathogen invasion. We further demonstrated that overexpression of TgATG13 induced formation of autophagosome. Our results not only shed light on the innate immunity mechanism of blood clam, but also provide clues on the prevention and control of shellfish diseases.

## 2. Materials and methods

### 2.1. Experimental animals and immune challenges

Healthy blood clams of the same size (shell length ~3.2 cm) were purchased from the Eighth Seafood Market of Xiamen, with an average weight of about 9.0 g. As previously reported, after 7-day incubation in filtered aerated seawater at 26°C, the foot muscle of each clam was injected with 10 μg LPS (Sigma, USA) and 20 μL live *V. alginolyticus* (2 × 10^8^ CFU/mL, pH 7.2), respectively, with PBS as the control (25). After injection, blood clams were maintained in the filtered aerated seawater for further experiments.

### 2.2. Molecular cloning of full-length TgATG13 cDNA

5'/3'- rapid amplification of cDNA ends-PCR (RACE-PCR) were used to obtain the full-length open reading frame (ORF) of TgATG13. The total RNA was extracted from hemocytes of three individual blood clams using TRIzol reagent (Invitrogen, USA) according to the manufacturer's instructions. SMARTer RACE 5'/3' Kit (Takara, Beijing, China) was used for RACE experiment. The partial sequence of TgATG13 gene was obtained from *T. granosa* transcriptome library in our laboratory. Gene-specific primers as shown in Table 1 were designed based on the partial cDNA sequence. PCR products were cloned into a pEASY-T1 vector and verified by DNA sequencing.

**Table 1:** Primers used in this study.

**Primer name**	**Sequence (5' to 3')**	**Application**
ATG13-R1	AAGGTTRAACCAATCGGCTCCWG	5′-RACE PCR
ATG13-R2	GATTTTCTCTCCCAATCTGGACT	5′-RACE PCR
ATG13-F1	AAATTTACCHWGGDGATCCMCAGTT	3′-RACE PCR
ATG13-F2	GAYCATTTYAAAAAAGACAAYAGTCC	3′-RACE PCR
M13F	GTAAAACGACGGCCAGT	Sequencing primer
M13R	CAGGAAACAGCTATGAC	Sequencing primer
ATG13-ORF-F	GGCGGAGGCGGATCAGGATCCATGTCAGGCTCCAAGTTA	ORF amplification
ATG13-ORF-R	CCCTCTAGACTCGAGTCAATCTGTCAGCGATTCAG	ORF amplification
ATG13-RT-F	CTGCTGGACTTGTGGAAAACG	qRT-PCR
ATG13-RT-R	GAGACCAACAACGCCAAAGC	qRT-PCR
Tg18S-F	CTTTCAAATGTCTGCCCTATCAACT	Internal control
Tg18S-R	TCCCGTATTGTTATTTTTCGTCACT	Internal control

### 2.3. Sequence alignment and phylogenetic tree construction

Bioinformatics analysis of ATG13 protein was achieved by several online tools and software. The TgATG13 protein domain was analyzed by InterPro (http://www.ebi.ac.uk/interpro). The physicochemical properties of ATG13 protein was analyzed by ExPASy PROSITE (http://www.expasy.ch/prosite). Prediction of the functional domain of LC3 and its corresponding tubulin motif was achieved by iLIR server (http://repeat.biol.ucy.ac.cy/iLIR). Prediction of protein secondary structure was achieved by CFSSP tool (http://www.biogem.org/tool/chou-fasman). Protein tertiary structure modeling was performed by SWISS-MODEL (http://swissmodel.expasy.org), using PyMOL (version 2.5.2) to visually adjust the prediction model. The ClustalW software was used for sequence alignment and similarity analysis of ATG13 homologs (26), while the phylogenetic tree of ATG13 homologs was constructed and analyzed by MEGA software and NJ (neighbor joining algorithm) algorithm with default setting (27).

### 2.4. Plasmid construction, cell culture, and transfection

The open reading frame (ORF) of TgATG13 was cloned into the pcDNA3.1-EGFP vector to construct pcDNA-EGFP-TgATG13 recombinant plasmid. HEK293T cells were maintained in DMEM containing 10% fetal bovine serum (Hyclone, USA). 24 h before the transfection, 6-well plates with glass slides were plated with 5 × 10^4^ cells per well. The cells were then divided into two groups and transfected with 0.5 μg pcDNA3.1-EGFP or pcDNA-EGFP-TgATG13 using Lipofectamine 2000 (Invitrogen, USA). In autophagy experiment, 0.5 μg GFP-LC3 plasmid (AntiHela, Xiamen, China) alone or with pcDNA3.1-HA-TgATG13 were transfected into HEK293T cells for 24 h. The positive control was treated with autophagy inducer rapamycin (100 nM) for another 24 h, followed by confocal microscopy analysis.

### 2.5. Confocal microscopy analysis

The HEK293T cells transfected with targeted plasmids were washed twice with PBS. 1 mL of 4% paraformaldehyde was added to each well for 10 min to fix cell and washing twice by PBS. 0.2% Triton X-100 was then added for 5 min and washed twice with PBS. The cells were stained by 1 μg/mL of DAPI (Solarbio, Beijing, China) and washed by PBS for three times. The subcellular localization of targeted proteins was observed by Leica SP2 confocal microscope (Leica, Germany).

### 2.6. Quantitative real-time PCR (qRT-PCR)

According to the cloned TgATG13 gene sequence, specific primers targeting TgATG13 were designed for qRT-PCR. 18S rRNA was used as the internal reference gene (Table 1). In tissue expression pattern analysis, blood clams were randomly selected to separately collect hemocytes, foot, visceral mass, adductor muscle, gill and mantle tissues. Total RNA was extracted and reversely transcribed from each tissue to detect the expression levels of TgATG13 gene using Rotor-Gene 6000 Real-time PCR system (Qiagen, USA). In stimuli challenging experiment, the blood clams were divided into three groups, injected with PBS, *V. alginolyticus* and LPS, respectively. Hemocytes of 3 blood clams from each group were collected at 6, 12, 24, and 48 h after injection, and total RNA was extracted and reversely transcribed into cDNA to detect the changes of ATG13 gene expression using qRT-PCR.

The qRT-PCR assay was performed with a system containing 5 μL 2 × SYBR Green Pro Taq HS Premix (Agbio, Changsha, China), 2 μL template, 0.8 μL of each primer (10 μM) and 3.2 μL of RNase free water. The programs for PCR amplification were carried out in the following setting 30 s at 95°C, 40 cycles at 95°C for 30 s, 60°C for 30 s and 72°C for 30 s. The qRT-PCR results were calculated using the comparative Ct (2^−ΔΔCt^) method.

### 2.7. Statistical analysis

Results are presented as the means ± standard deviation of triplicate experiments. Differences between the groups were statistically tested using student's *t*-test or one-way analysis of variance. A difference was considered significant when *P* < 0.05.

## 3. Results

### 3.1. Molecular cloning and characterization of TgATG13

**Figure 1:**
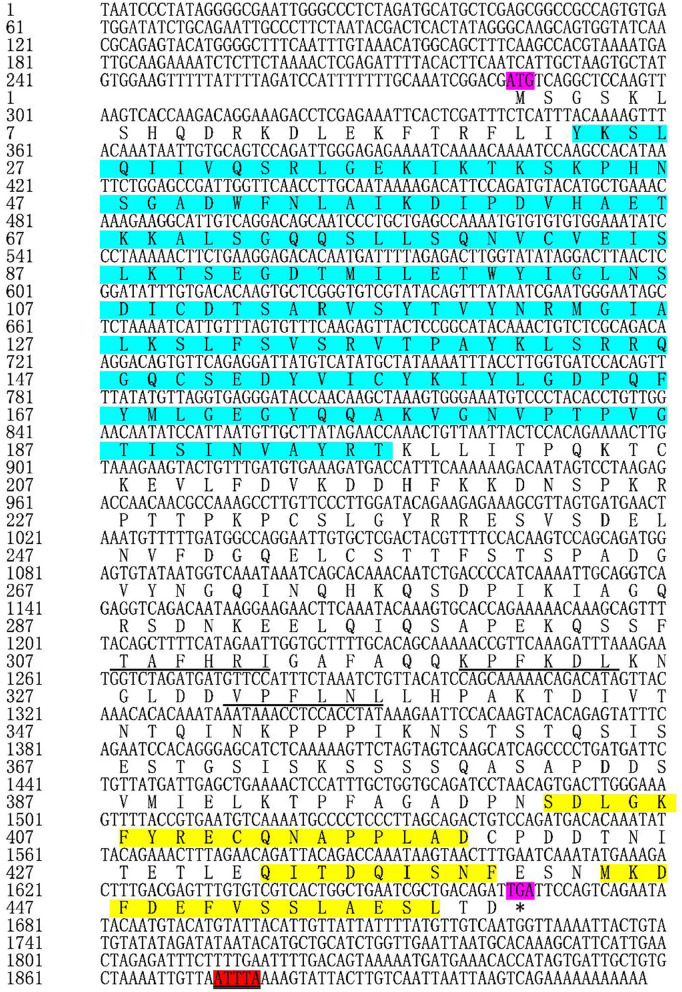
Nucleotide and deduced amino acid sequences of TgATG13. The start codon (ATG) and stop codon (TGA) are labeled purple. The amino acids (23–196) that form the conserved HORMA domain are labeled blue. The typical ATTTA motif was marked red. The action domains of LC3 are marked by underline and the corresponding three tubulin motifs are labeled yellow. * represents the stop codon.

Based on the transcriptome data we previously collected, we obtained full length *TgATG13* cDNA sequence using RACE technique. The TgATG13 nucleotide and deduced amino acid sequences are shown in Figure 1. The TgATG13 gene had a 1,383 bp ORF encoding a protein of 460 amino acid residues. The 5' -untranslated region (UTR) was 283 bp in length, and the 3' -UTR contained 252 nucleotides ended with a typical ATTTA motif and poly (A) tail (Figure 1). The molecular weight of deduced TgATG13 protein is about 51.11 kDa, with a pI of 6.05. The instability coefficient and Grand average of hydropathicity (GRAVY) of TgATG13 were 39.77 and −0.562 respectively, indicating that TgATG13 protein was a stable hydrophilic protein.

Through the CD Search function of NCBI website, it was found that TgATG13 protein contained a conserved HORMA domain (residues 23–196), which plays an important role in eukaryotic cell cycle regulation. The result also showed that TgATG13 protein was a member of the phosphorylation protein family and possibly involved in the transport of cytoplasm to vacuole (Cvt). The iLIR web server predicted that the ATG13 gene contained three autophagy-related protein LC3 domains and three corresponding tubulin motifs.

**Figure 2:**
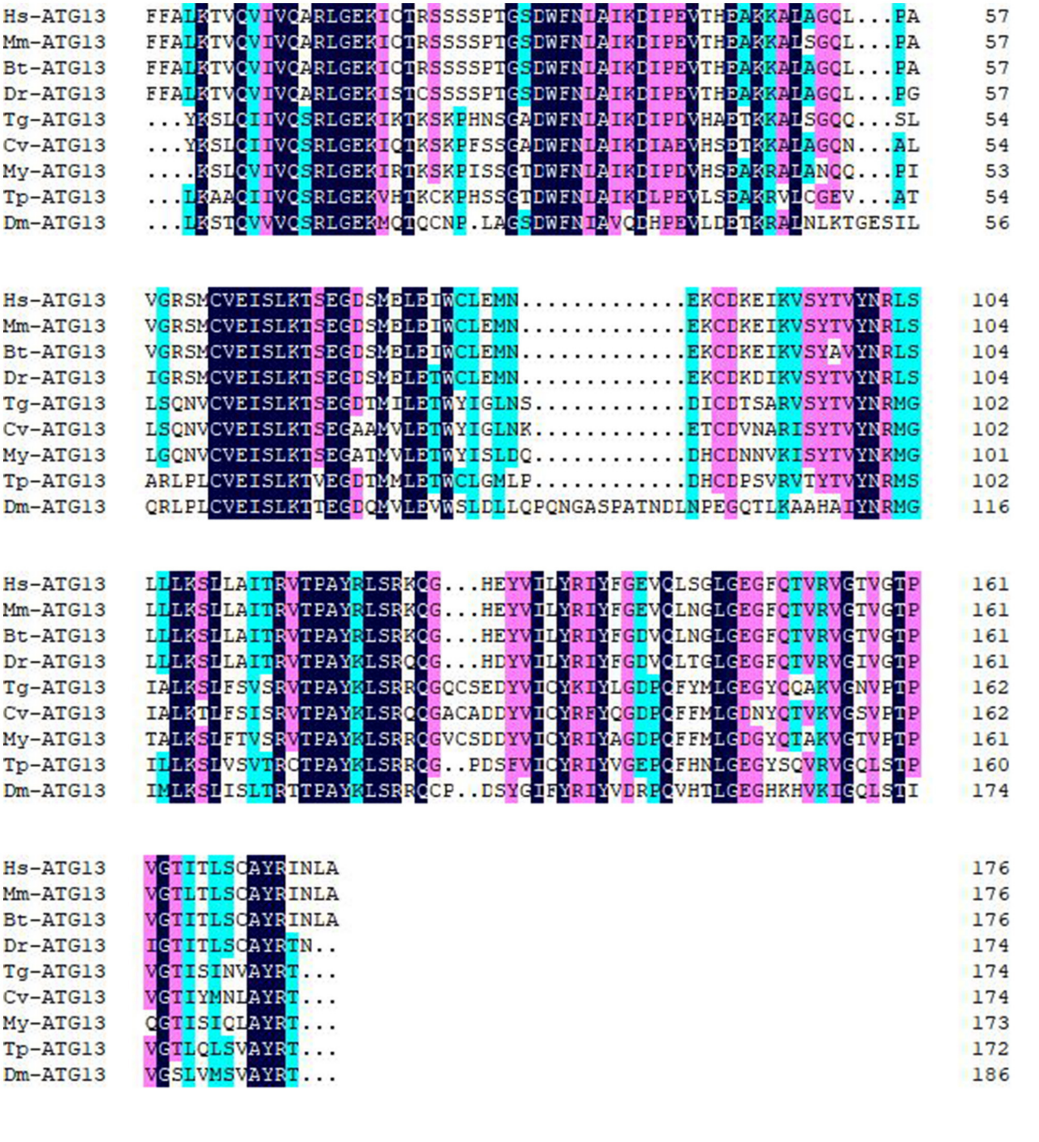
Amino acid sequence alignment of ATG13 HORMA domains from blood clam and other species. Amino acid sequence alignment was performed ClustalW software. Hs, *Homo sapiens*; Mm, *Mus musculus*; Bt, *Bos Taurus*; Dr, *Danio rerio*; Tg, *Tegillarca granosa*; Cv, *Crassostrea virginica*; My, *Mizuhopecten yessoensis*; Tp, *Thrips palmi*; Dm, *Drosophila melanogaster*.

**Figure 3:**
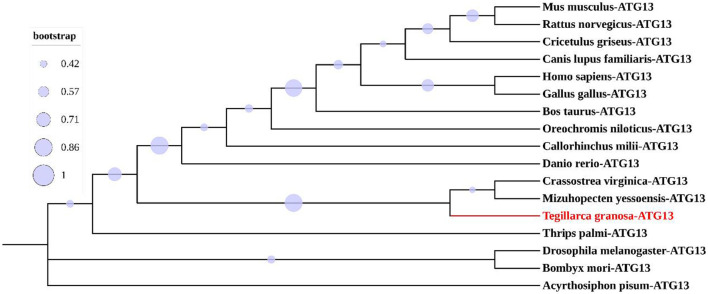
Phylogenetic analysis of ATG13 HORMA domains from different species.

Multiple sequence alignment with conserved HORMA domains of ATG13 homologs was shown in Figure 2. TgATG13 had the highest identity (79.31%) with *Crassostrea virginica* ATG13. The HORMA domain of TgATG13 was further analyzed with the representative ATG13 homologs through a phylogenetic study using the neighbor-joining method (Table 2). Phylogenetic analysis indicated that TgATG13 clustered strongly with the *C. virginica* ATG13 and *Mizuhopecten yessoensis* ATG13 with a high bootstrap value (Figure 3).

**Table 2:** The GenBank access numbers of ATG13 homologs used in multiple sequence alignment and phylogenetic tree analysis.

**Species name**	**GenBank access number**
*Homo sapiens*	NP_001136145.1
*Mus musculus*	NP_001342348.1
*Bos taurus*	NP_001070280.1
*Danio rerio*	NP_956727.1
*Crassostrea virginica*	XP_022321200.1
*Mizuhopecten yessoensis*	XP_021375794.1
*Thrips palmi*	XP_034254790.1
*Drosophila melanogaster*	NP_649796.1
*Rattus norvegicus*	NP_001258141.1
*Cricetulus griseus*	XP_016820706.1
*Gallus gallus*	XP_015142708.1
*Oreochromis niloticus*	XP_005460698.1
*Callorhinchus milii*	AFP01762.1
*Acyrthosiphon pisum*	XP_029343894.1
*Bombyx mori*	XP_004924339.1
*Canis lupus familiaris*	XP_022261189.1

**Figure 4:**
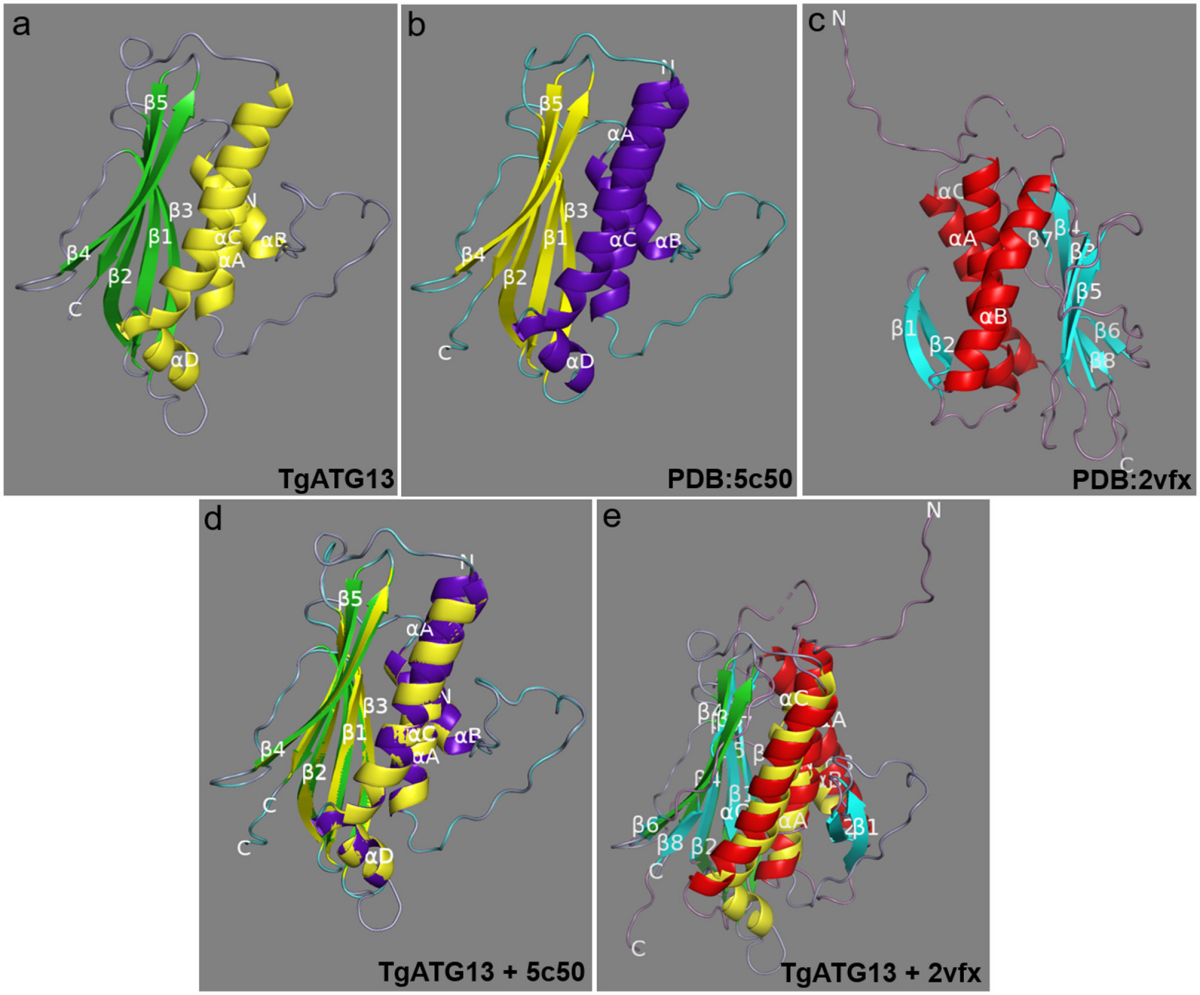
Molecular structure of the TgATG13 HORMA domain. **(a)** Predicted structural model of the N-terminal HORMA domain of TgATG13. **(b)** Reference model human ATG101-ATG13 complex HORMA domain (PDB ID: 5C50). **(c)** Spindle checkpoint protein Mad2 dimer (PDB ID: 2VFX). **(d)** The 3D image of the N-terminal HORMA domain of TgATG13 was superimposed on the reference model ATG13 (PDB ID: 5C50). **(e)** The 3D image of the N-terminal HORMA domain of TgATG13 was superimposed on the Mad2 dimer (PDB ID: 2VFX). The α-helix and β-sheet of the reference model are expressed in purple and yellow, respectively. The α-helix and β-sheet of the TgATG13 HORMA domain model are expressed in yellow and green. The α-helix and β-sheet of the Mad2 dimer model are expressed in red and light blue.

The amino acid sequence of TgATG13 HORMA domain was online modeled on SWISS MODEL website, and it was found that its similarity with human ATG101-ATG13 complex HORMA domain (PDB ID: 5C50) was up to 56.63% (Figures 4a,b). PyMOL software was further used to extract the B chain of 5C50, and the alignment function was used to compare the structures of two ATG homologs. The RMSD of the two structures was 0.078. The superposition of the two structures was shown in Figure 4d. The coincidence degree of the two structures was high in the α-helix structure, but there was an incomplete coincidence difference on the β-sheet. In addition, the predicted TgATG13 HORMA structure was also similar to the spindle checkpoint protein Mad2 dimer (PDB ID: 2VFX) (Figures 4a, c). About half of the ordered part of the TgATG13 HORMA domain could be superimposed on the structure of Mad2 (Figure 4e).

### 3.2. Subcellular location of TgATG13

**Figure 5:**
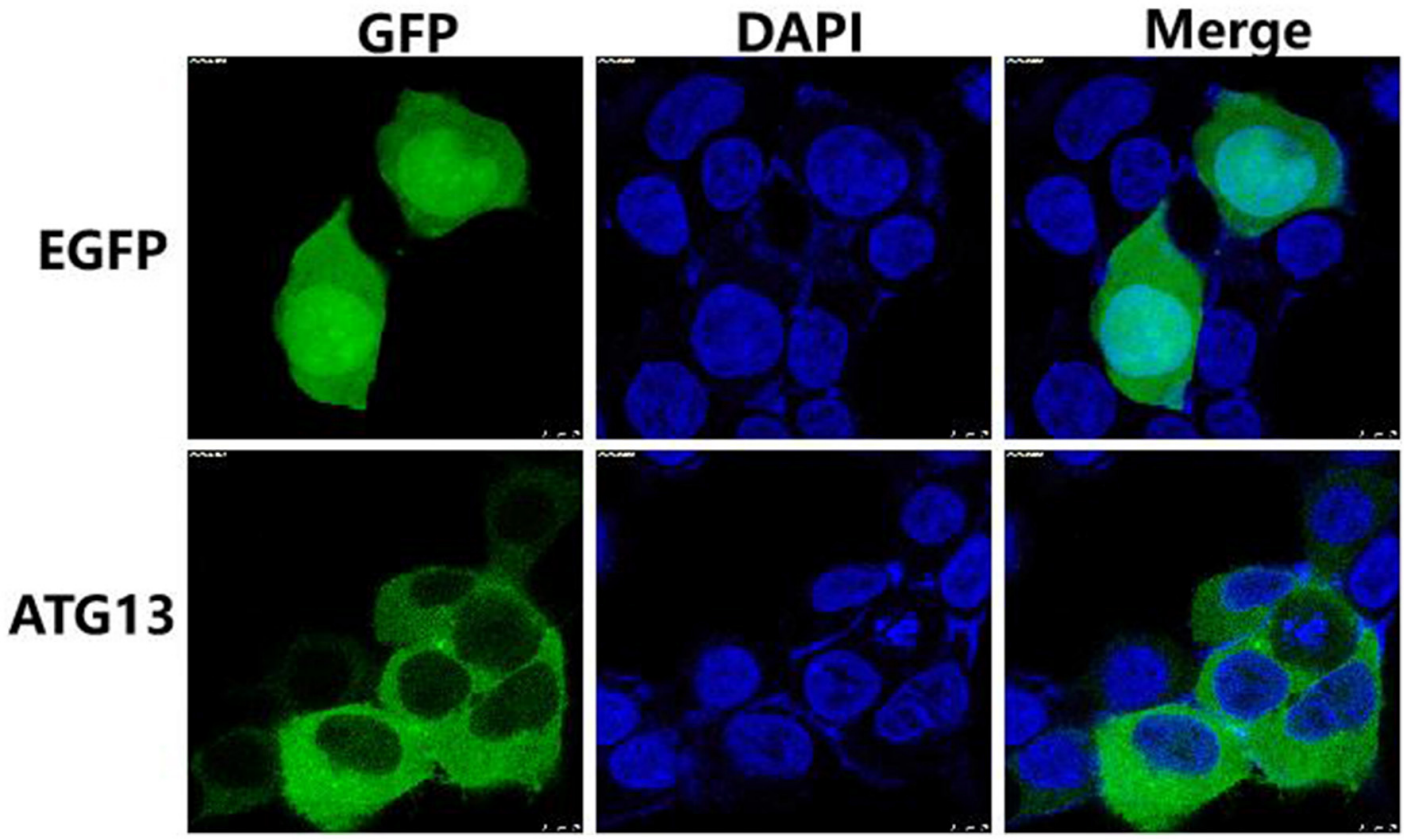
Subcellular localization of TgATG13 in HEK293T cells. HEK293T cells were transfected with pcDNA3.1-EGFP or pcDNA3.1-EGFP-TgATG13 for 24 h. DAPI was used to stain cell nuclei. Scale bar = 7.5 μm.

In order to determine the subcellular location of TgATG13, HEK293T cells were transfected with plasmids encoding EGFP alone and EGFP-TgATG13, respectively. The subcellular expression pattern of these two proteins was determined by confocal microscopy. In the control cells transfected with pcDNA3.1-EGFP, GFP fluorescence was expressed ubiquitously in the cell. The TgATG13 protein was mainly located in the cytoplasm, which expression pattern was similar to its human ATG13 homolog (Figure 5).

### 3.3. Tissue distribution of TgATG13

**Figure 6:**
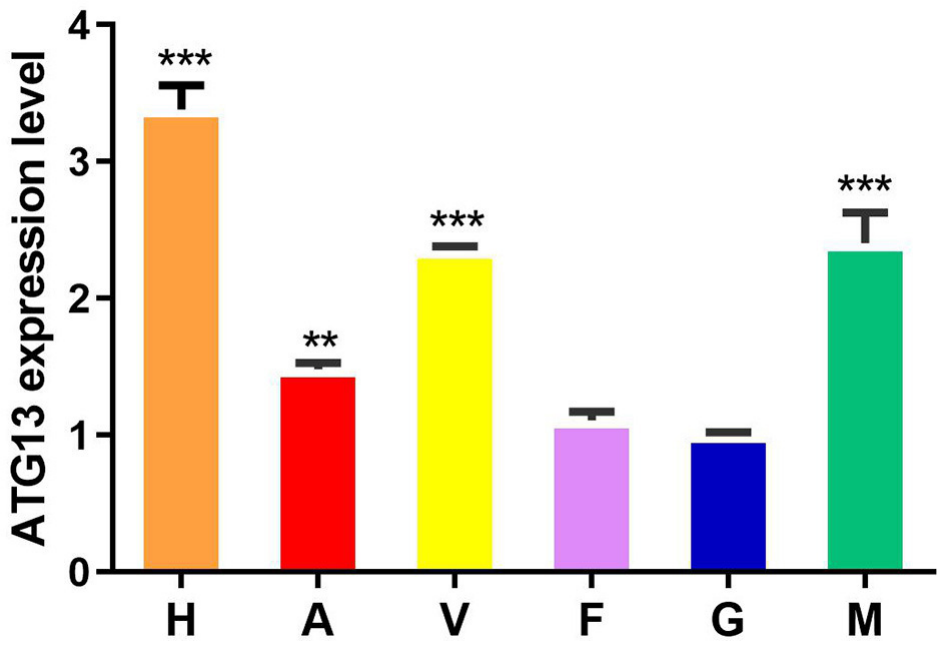
Expression analysis of *TgATG13* gene in different tissues of blood clam. The expression level of *TgATG13* gene in different tissues was investigated using qRT-PCR with 18S rRNA as the internal control. H represents hemocytes, A represents adductor muscle, V represents visceral mass, F represents foot, G represents gill, and M represents mantle. Error bars represent the mean ± S.D. (*n* = 3). ^**^ *P* < 0.01, ^***^ *P* < 0.001.

The qRT-PCR results showed that *TgATG13* gene was ubiquitously expressed in all tissues examined with different expression levels, including hemocyte, gill, foot, mantle, adductor muscle and visceral mass (Figure 6). The expression level of *TgATG13* was lowest in gill. *TgATG13* had a 3.38-fold expression in hemocytes than that in gill. Mantle, visceral mass and adductor muscle also showed significantly higher *TgATG13* expression levels compared to gill.

### 3.4. Temporal expression pattern of TgATG13 after stimulation

**Figure 7:**
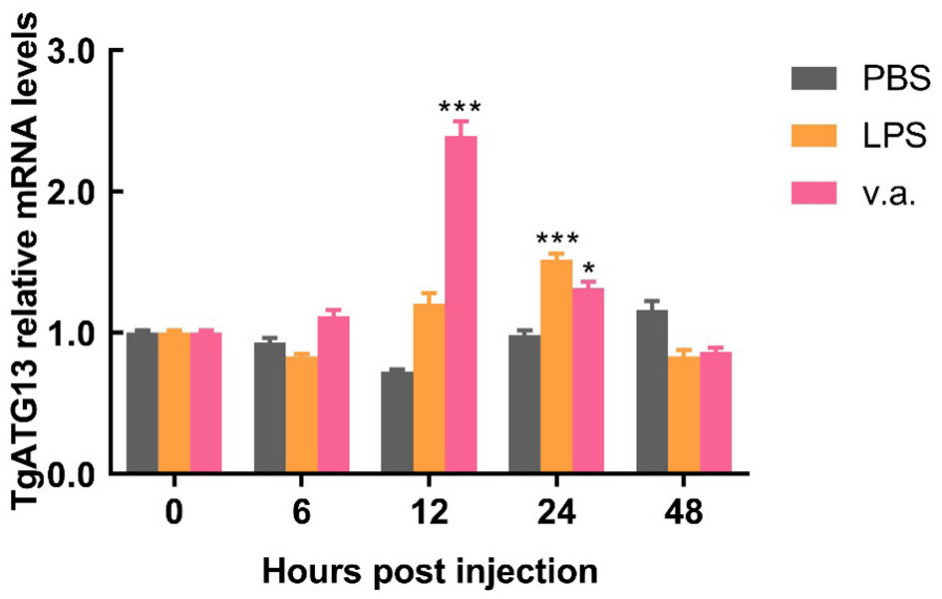
Temporal expression pattern of the transcript of TgATG13 in blood clam hemocytes after challenges. The expression levels of TgATG13 mRNA in different time points post LPS and *V. alginolyticus* challenges were investigated using real time PCR assay with 18S rRNA as the internal control. Error bars represent the mean ± S.D. (*n* = 3). ^*^P < 0.05, ^***^ *P* < 0.001.

The blood clam was treated with LPS or *V. alginolyticus* by foot injection, and the expression of *TgATG13* gene in hemocytes was detected by qRT-PCR at different time points after injection. Compared with PBS group, both LPS treatment group and *V. alginolyticus* treatment group showed enhanced TgATG13 expression level in 24 h post injection. LPS-stimulated TgATG13 expression reached its peak level at 24 h with a 1.5-fold increase compared with control (Figure 7). The expression of *TgATG13* in *V. alginolyticus* treatment group was also activated and reached a maximum value at 12 h with a 2.3-fold increase (Figure 7). These results demonstrated that TgATG13 could respond robustly to the pathogen stimulation, suggesting that TgATG13 may be involved in innate immune regulation of blood clam against pathogen invasion.

### 3.5. TgATG13 participates in autophagy

**Figure 8:**
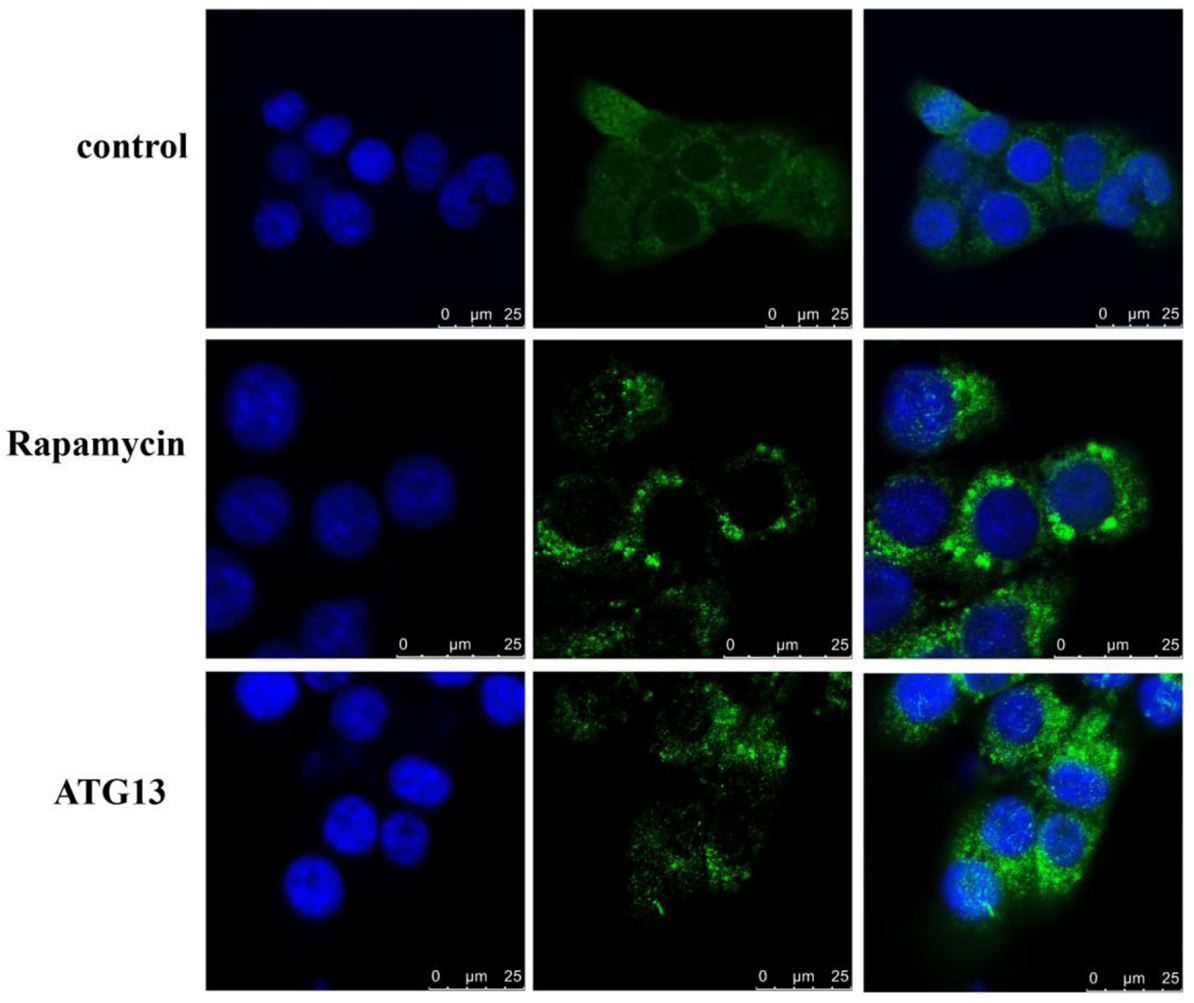
Confocal fluorescence microscopy of autophagy puncta. HEK293T cells were transfected with GFP-LC3 plasmid alone or with pcDNA3.1-HA-TgSTING plasmid for 24 h. For positive control, 100 nM rapamycin was added into cells for another 24 h. DAPI was used to stain cell nuclei. Scale bar = 25 μm.

In order to investigate whether TgATG13 is involved in autophagy, we transfected GFP-LC3 plasmid into HEK293T cells to detect the autophagic puncta formation. We found that in the positive control group, in which the autophagy inducer rapamycin was added, a large number of autophagy puncta was observed (Figure 8). Similarly, the cells expressing TgATG13 protein also showed a significant increase of autophagy puncta, indicating that TgATG13 promoted the production of autophagosomes, and therefore participated in autophagy.

## 4. Discussion and conclusion

Currently, some progress has been made in the research on the role and molecular mechanism of ATG13, which mainly focused on mammalian cells, with few studies on shellfish. In this study, the full-length cDNA sequence of TgATG13 gene from blood clam was obtained. Amino acid sequence analysis demonstrated that TgATG13 protein contained three LC3 action domains and three corresponding microtubule protein action motifs. Studies have shown that autophagy-related protein LC3 is vital in the process of autophagosome membrane elongation and maturation, and is associated with microtubule proteins through LC3 interaction region (LIR) (28, 29). Therefore, it is possible that microtubule proteins with transport, regulation and migration may be related to autophagosome transport in blood clam.

There have been some studies investigating the aforementioned speculation. In yeast and mammals, several members of the ATG1/ULK1 core complex have been proved to interact with the autophagy core protein LC3/ATG8 through the LIR element. By connecting the ATG1/ULK1 complex with the autophagy membrane, the LIR element can not only concentrate the kinase complex on the amplified autophagosome, but also target it to the lysosome for degradation, so as to control the autophagy flux (30). Moreover, amino acid sequence analysis showed that TgATG13 protein had a highly conserved HORMA domain at its N-terminal, which was highly similar to its homologs from other species. Three-dimensional structure of TgATG13 protein was predicted by homology modeling, and it was found that about half of the sequential part of the structure could be superimposed on the structure of Mad2, which was similar to the related research of human and yeast. Among them, the HORMA domain at the N-terminal of ATG13 in the yeast has an assumed phosphate binding site to recruit membrane protein ATG9 and autophagy PI3K kinase complex (31).

Based on the phylogenetic analysis of ATG13 homologs from representative species, ATG13 was highly conserved during evolution. TgATG13 had the highest homology with *C. virginica* ATG13, with the similarity rate at 79.31%. Phylogenetic analysis also demonstrated that TgATG13 protein was clustered closely with ATG13 homologs from shellfish, such as *C. virginica* ATG13 and *M. yessoensis*, far with the homologs from arthropods such as insects. However, TgATG13 was divergent from other branches of fish and mammals in vertebrates.

Furthermore, qRT-PCR results showed that the expression levels of *TgATG13* gene varied in different tissues of blood clam. The *TgATG13* gene was highly expressed in hemocytes, which is reasonable since hemocytes played a central role in the host immune defense of bivalve mollusks. It was also reported that hemocytes were involved in the regulatory mechanism of phagocyte activation. For instance, Mao et al. (32) isolated granulocytes and transparent cells from the hemocytes of *C. a gigas* using flow cytometry. Granulocytes are in charge of immune prevention and control, and have strong phagocytosis. Moreover, transcription factors such as ELK, HELT and Fos are expressed in granulocytes. Subsequently, *V. alginolyticus* or LPS were injected into the foot muscle to stimulate the immune response of blood clam. *V. alginolyticus* and LPS stimulation were found to enhance the expression of *TgATG13* gene in the hemocytes through the qRT-PCR test. *V. alginolyticus* infection can stimulate autophagy in *C. gigas*, and the use of Cdc42 inhibitors significantly reduced the phagocytosis of blood cells, caused apoptosis and decreased the bactericidal ability (32). Therefore, we assume that after the infection of *V. alginolyticus*, the blood cells enhance the expression of *TgATG13* gene and stimulate autophagy for self-protection.

Aiming to confirm that TgATG13 is involved in autophagy, we transiently transfected GFP-LC3 plasmid to track the autophagy process. Laser confocal microscopy analysis demonstrated that TgATG13 is involved in the formation of autophagosomes. This phenomenon is consistent with the study conducted by Hegedüs et al. (33). In their research, they found that ULK1 polyprotein complexes composed of ATG13, FIP200 and ATG101 played a key role in inducing autophagy in Drosophila autophagy model. On one hand, ATG13 and FIP200 were indispensable in targeting ULK1 complexes to the autophagy bodies, as well as the stability and kinase activity of ULK1. On the other hand, binding ATG101 to ULK1 complexes through the N-terminal of ATG13 was necessary for the self-phosphorylation of ULK1.

In summary, we have obtained the full-length cDNA sequence of TgATG13 gene. TgATG13 protein is highly conserved with its homologs from other species. The expression level of *TgATG13* was robustly increased after stimulations of both *V. alginolyticus* and LPS. Fluorescence confocal microscopy further showed that TgATG13 promoted the production of autophagosome. Our study demonstrated that TgATG13 was involved in the immune regulation of blood clam during pathogen invasion, deepening our understanding of the innate immune mechanism of blood clam.

## Data availability statement

The original contributions presented in the study are included in the article/supplementary material, further inquiries can be directed to the corresponding authors.

## Author contributions

MC, ZL, and QW contributed to conception and design of the study. YW and ZL organized the database. YW and GL performed the statistical analysis. YW, MC, ZL, and QW wrote the first draft of the manuscript. All authors contributed to manuscript revision, read, and approved the submitted version.
